# Biometric Analysis of Cranial and Somatic Features in the Pannonian Root Vole

**DOI:** 10.3390/ani11020576

**Published:** 2021-02-23

**Authors:** Ivan Baláž, Filip Tulis, Michal Ševčík

**Affiliations:** Department of Ecology and Environmental Sciences, Faculty of Natural Sciences, Constantine the Philosopher University in Nitra, Trieda A. Hlinku 1, 949 74 Nitra, Slovakia; ftulis@ukf.sk (F.T.); msevcik@ukf.sk (M.Š.)

**Keywords:** *Alexandromys oeconomus mehelyi*, skull, lower jaw

## Abstract

**Simple Summary:**

The aim of this paper is to biometrically analyse the cranial and somatic features of the Pannonian root vole in Slovakia and link body weight to selected cranial features. Somatic features indicate that specimens caught in Slovakia belong to the largest sub-species *oeconomus*. Overall, our results showed sexual dimorphism of all the somatic features observed in adult specimens, while the effect of seasonality was only seen in the average length of the body and tail. Thus, mean body and foot length appear stable in determining features despite the seasons. This study evaluated 25 skull and lower jaw measurements, representing the largest number of evaluated characteristics among Slovakia’s Pannonian root vole population. Linear regression of the weight and any of the three craniological features AMd, AMdm, and LCr is recommended in order to predict weight directly from them. This analysis is useful as a non-invasive method for analysing skeletal food remains that have been found in raptors and owls. Using the correlation between weight and body length makes it possible to analyse the Pannonian root vole population structure in greater detail, such as to classify specimens into age cohorts.

**Abstract:**

The Pannonian root vole *Alexandromys oeconomus* ssp. *mehelyi* represents a rare glacial relict, whose occurrence is nowadays bound to several areas in Europe. Four somatic and 25 craniological features were analysed, based on 355 measured specimens. Sex is a significant factor affecting the average value of all four somatic features, where all of them achieve higher values in males than in females. While body length and tail length were also affected by seasons, body weight and the length of the hind foot were stable features present across the seasons. In cranial features, the largest variability in the adult population is characterised by neurocranium breadth (LaN), total length of the cranial base (LB), and skull (LCr); whereas the smallest variability of the cranial dimensions is reflected in the values of the greatest palatal breadth (PS) and postorbital breadth (Io). Calculating the weight from cranial remains may be used to estimate the size of the prey and to determine vole biomass consumed by predators, such as raptors, highlighting the utility of studying feeding ecology.

## 1. Introduction

The key to protecting and safeguarding the vitality of rare species is a detailed knowledge of their biology and ecology [1]. The Pannonian root vole, *Alexandromys* (*Microtus*) *oeconomus* (Pallas, 1776) ssp. *mehelyi* Éhik, 1928, [2] is one of the scarcest and most endangered mammalian species in Slovakia, a subspecies whose range had originally covered continually much of the Holarctic realm. In addition to Slovakia, it is still native locally in Austria and in three isolated locations in Hungary [3]. As mentioned above, this glacial relict of the Pannonian Plain is today quite rare—the threat status in Europe is Least Concern (IUCN) and in Red list of plants and animals of Slovakia Nature Conservation is rated as EN—an endangered species [4]. It is currently designated in Annex II and IV of the Habitat Directive (92/43/EEC) as a Species of European Interest.

In Slovakia, the Pannonian root vole lives only in specific habitats, which are the remnants of former wetlands that have become more or less filled in or otherwise degraded. These comprise either parts of water catchment areas, including oxbow lakes, or what still exists of the Danubian Plain’s surface river network. In these habitats, the species finds appropriate trophic and local environmental conditions (waterlogged habitats with suitable vegetation—reed *Phragmites* sp. and sedges *Carex* sp.) [5]. The Pannonian root vole’s current distribution in Slovakia is marked by landscape development in the centuries since the Ice Age [6]. Over the last 150–200 years, there has been significant water management of the Danube’s tributaries and the lower catchment areas of the rivers flowing from the Carpathian Mountains [5]. There have also been changes in land use, such as agricultural engineering, drainage, and land improvement [7], transforming the region into a cultural steppe and fragmenting the habitat where Pannonian roles voles had once thrived. These changes in land use have caused the generic variability of the species to decline, creating isolated individual populations with no significant opportunities to interact with each other [8].

There are four distinct mitochondrial DNA (mtDNA) clades among root voles [2]. The Central European clade covers southern Scandinavia, the Netherlands, Poland, Lithuania, Hungary, and south-western Slovakia, while the Northern European clade comprises northern and eastern Scandinavia, Belarus, and Russia in Europe; the Central Asian clade Siberia and the western part of the Russian Far East and the Beringian clade the Chukchi and Kamchatka Peninsulas, Kuril Islands, Alaska, and western Canada. There are twenty-five subspecies recognised by Wilson et al. [2], with the nearest subspecies being *A. oeconomus stimmingi* in Poland, eastern Germany, Lithuania, Latvia, Belarus, Ukraine, and in central European Russia; and *A. oeconomus arenicola* in the Netherlands.

Morphometric (craniometric and somatometric) data about the Pannonian root vole from both Slovakia [8,9,10] and other locations where the subspecies occurs ([11] in Austria; [12,13] in Hungary) are available. Krištofík and Stollmann [14] have documented differences in the weight of species in Slovakia depending on the season of the year. Kratochvíl and Rosický [9] considered the hind foot and diameter of the northern Pannonian vole’s eye to be reliable determinants, well distinguishable from other voles occurring in our territory. Rácz et al. [13] monitored the historical relationship between regional subspecies populations in Central Europe based on the analysis of morphological similarities of skulls and lower jaws. Comparison of lower jaws and skulls indicate that root vole populations form four regional clusters in Hungary.

Analysing the relationship between rodent, including some root vole sub-species, body weight and their cranial or body traits has been a widely used approach in order to estimate body weight and biomass of mammals consumed by predators [15,16,17,18,19,20,21,22,23,24,25,26]. Borowski et al. [25] investigated the relationships between the biometrics of cranial traits and the body weight of *Alexandromys oeconomus stimmingi*, while Balčiauskas and Balčiauskiené [27] estimated body weight from 26 cranial and pelvic features of *A. oeconomus stimmingi*. Such craniological features that were not destroyed and had managed to be preserved even after consumption by predators are especially significant. These can be used to calculate and estimate weight of voles based on cranial measurements, such as Canova et al. [28] who suggested regression to estimate the weight of the skulls and jaws of small mammals.

This paper seeks to biometrically analyse the cranial and somatic features of the Pannonian root vole in Slovakia and link body weight to selected cranial features. Morphometric analysis of the Pannonian root vole has not yet been performed on the basis of such a huge material (434 specimens) and not so many cranial features (25 skull and lower jaw measurements) in this subspecies have been evaluated. We tested the differences in the observed cranial features between adult males and females of the Pannonian root vole as well as the effect of the season on these measurements. The relationship between weight and craniological features was also analyzed. We hypothesis that the sex and season does not affect the measurements of the observed features of the adult species. Such analyzes and evaluations have not yet been performed on this rare subspecies.

## 2. Materials and Methods

Pannonian root vole specimens were obtained from locations in the Danubian Plain and in the Hronská pahorkatina highlands in south-western Slovakia. The physiognomy of this region’s landscape has been transformed by the action of the Hron, Žitava, and Váh, whose currents slow when the rivers reach the Danubian Plain. Here they start meandering to create the wetland habitats that provide the Pannonian root vole with the optimal conditions to thrive. The habitats are formed by stands of common reeds (*Phragmites*) in the stagnant waters and swamps, with sedges (*Carex*) and cattails (*Typha*) also growing at spots. Trees found in this area are the brittle willow (*Salix fragilis*) and groves of grey poplars (*Populus* × *canescens*).

**Figure 1:**
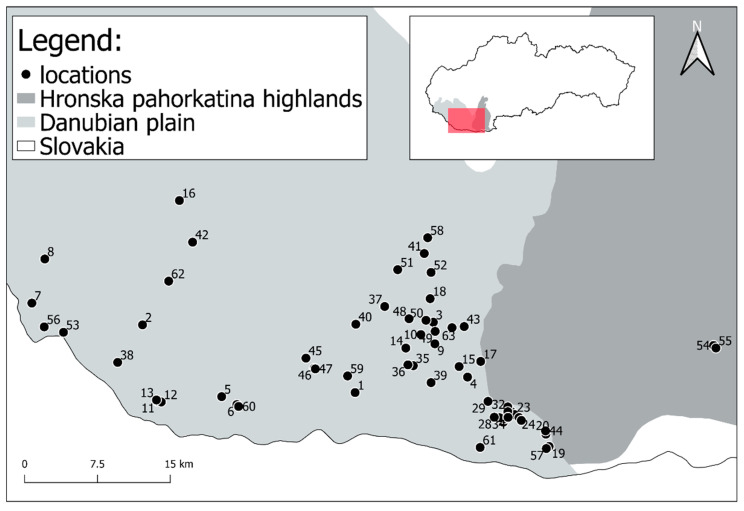
Localities of trapping Pannonian root vole.

Voles were caught in snap traps until 2004 (from 1975), but since that year wooden traps (trap dimensions: 200 × 80 × 100 mm, length × width × height) have been used that do not kill them. Snap traps were checked once a day while live animal traps twice a day and left closed during the night. The traps were filled with bait—a mixture of cereals, apples, and mealworms. The line method was employed to catch voles and 50 capture points within ten metre distances one to each other were set up. The captured vole specimens came from 63 locations in south-western Slovakia and so the analysis also included animals that had been caught in snap traps or happened to have accidentally died in the live traps (Figure 1, Table S1).

The sex of each specimen was determined and they were divided into three age groups: juvenile, sub-adult, and adult. The sexual activity of the adult specimens was also monitored (testes in scrotum and open vagina). Sub-adult specimens were defined as similar-sized individuals as adults, but not sexually active. Juveniles are smaller than sub-adults and have juvenile fur. However, age and gender were not determined in some cases, especially in specimens that had been captured longer ago in the past. Altogether, 434 specimens—314 animal caught in snap traps and 120 in live traps—were analysed over a period of 37 years (Table S1). Some specimens were analysed only for somatometry, some only for craniometry and some for both (Table 1).

Somatic features measured were: weight “W” in grams, body length “LC” from the head at the beginning of the rhinarium to the root of the tail, the length of the tail itself “LCd” from the root to its end without the end fur, and the length of the hind foot “LTP” from where the heel joint protrudes to the end of the longest toe without measuring the claw. We have obtained somatic features from all animals and analysed skulls only from animals captured in snap traps or accidentally died in live traps.

**Figure 2:**
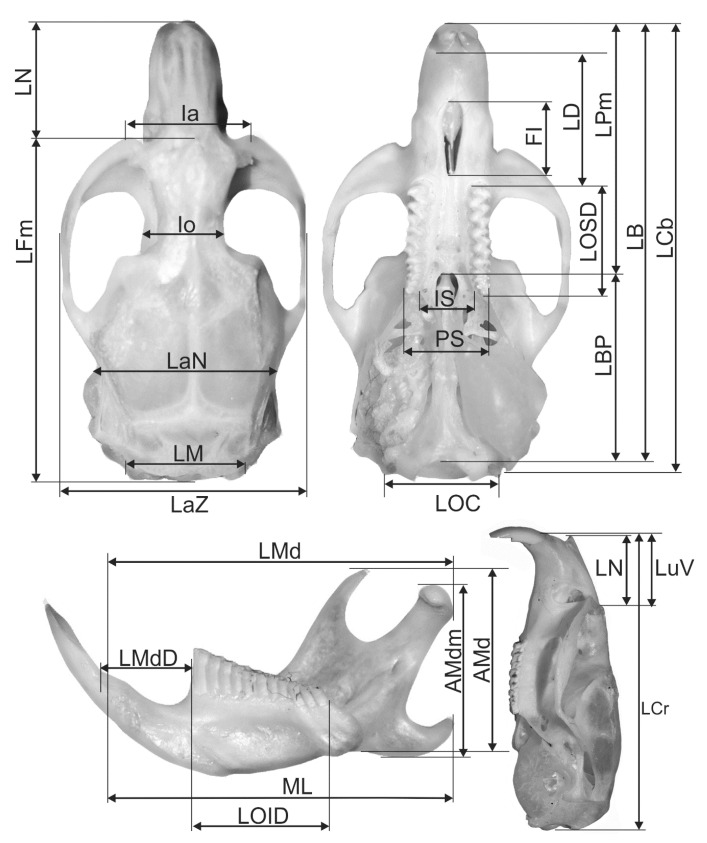
Measured skull and lower jaw dimensions of Pannonian root vole.

All skulls were cleaned with the aid of carnivorous beetles of the genus *Dermestes*. Paired features were always measured on the right side of the skull and jaw. The following cranial and jawbone features were monitored (according to Komosa et al. [29], Borowski et al. [25], Figure 2): LCr—total length of skull (*Akrokranion*—*Prosthion*), LCB—condylobasal length (*condylus occipitalis*), LB—total length of the cranial base (*Basion*—*Prosthion*), LBP—basal-palatal length (*Basion*—*Staphylion*), LPm—median palatal length (*Staphylion*—*Prosthion*), LFm—median frontal length (*Akrokranion*—*Nasion*), LuV—upper length of the *viscerocranium* (*Nasion*—*Prosthion*), LN—length of the nasals (*Nasion*—*Rhinion*), LaZ—zygomatic breadth (*Zygion*—*Zygion*), Ia—breadth across the supraorbital processes, Io—postorbital breadth (*Frontostenion*—*Frontostenion*), LOSD—length of the tooth row in the maxilla, LD—length of the diastema, LaN—neurocranium breadth (*Euryon*—*Euryon*), LM—length of the nuchal crest, LOC—breadth of occipital condyles, IS—breadth of incisive bone, PS—greatest palatal breadth, FI—length of *foramen incisivum*, LMd—total length of mandibula at *processus articularis* (*longitudo mandibulae*), AMd—coronoid height of mandibula (*altitudo mandibulae*), AMdm—maximum height of mandibula excluding coronoid process (coronoid process), LOID—length of mandibular tooth row (*longitudo ordinis inferioris dentium*), ML—mandible length excluding incisors, LMdD—length of mandibular diastema (Figure 1). All somatic and cranial features were measured with an electronic slide calliper to an accuracy of 0.1 mm. A 7× magnifying glass was used to obtain a detailed description of the dimensions.

Biometric data were processed by descriptive statistics to obtain the mean, standard deviation (SD), range, coefficient of variation (CV), and population size (*n*). We quantified the correlation between the examined somatic features using Pearson linear correlation. Two-way ANOVA monitored the impact of seasonality (spring, summer, and autumn) on the average log value of the four observed somatic features in adult specimens and it was also used to measure the effect, ascertain gender, and define interactions between them, while the Tukey HSD method determined post-hoc differences among the combination of factors. Due to the small amount of data captured during the winter, no winter data was included in the analysis. Likewise, because of the lack of winter and spring data, the effect of season and gender on somatic features was not analyzed in sub-adults. The t-test was employed for sub-adult specimens to monitor solely the difference in weight between summer and autumn. The lack of data (*n* = 23) led us not to analyse somatic feature in juvenile voles. Linear regression was used to determine the relationship between log weight and individual independent variables. To obtain a general picture of this relationship, the entire sample was entered into the calculation, without grouping them by gender, age, season, and location. Because of missing data in the dataset, not every analysed feature came from all 124 specimens in the sample. The normal distribution of values was tested using the Shapiro-Wilk test of normality. Bartlett’s test was used to determine the homogeneity of variances. Data outside the normal distribution were log-transformed to improve their normality. Statistical significance was tested at the levels of *p* < 0.05; *p* < 0.01, *p* < 0.001.

All statistical analyses were performed in the R environment [30].

## 3. Results

### 3.1. Biometric Analysis of the Somatic Features of Pannonian Root Voles

Analysing the somatic features in the groups, it was found that body weight increased as tail length became longer, while the length of the body was characterised by relatively low variability and the length of the hind foot had the least variability (Table 2). These points were confirmed in all of the analysed Pannonian root vole groups that had been divided by age and gender. The tail was 45.64% of the average length of an adult body (46.22% in males and 45.12% in females). In sub-adult specimens, the ratio of the average tail length to the average body length was 45.92% (46.82% in males, 45.3% in females). All of the somatic features in both the sub-adult and adult population reached higher mean values in males than females, except for body weight, which achieved higher values in females in the sub-adult age category (Table 2).

We noticed demonstrable sexual dimorphism in the weight of adult Pannonian root voles, yet there was contrarily no such effect observed for the season when they were captured and in the interaction between gender and seasonality. No interaction between these factors was documented. Tukey’s test for post hoc analysis showed an identifiable difference in body length of adult animals between spring and autumn (*p* = 0.004). The influence of season and gender on tail length was also evident. However, no interaction between them was proven. Subsequent Tukey’s post-hoc testing showed noticeable differences in body length of adult animals between spring and autumn (*p* < 0.001) and between spring and summer (*p* < 0.001). The effect of gender on the hind feet of Pannonian root voles was significant, while, on the other hand, no impact was documented of seasonality and the interaction between sex and seasonality (Table 3). A decrease was recorded in the weight of sub-adult specimens between those captured in summer and in autumn (summer: *n* = 26; average = 28.1 g autumn: *n* = 26; average = 24.4 g; *t*-test = 3.37, *p* < 0.001).

We also recorded a strong positive correlation between the Pannonian root vole’s body weight and its combined body and tail length, and likewise between its body and tail length. A medium correlation was also observed between the Pannonian root vole’s hind foot length and body weight and its tail and there was a weak correlation between the length of the hind foot and body length (Table 4).

### 3.2. Biometric Analysis of the Craniological Features of Pannonian Root Voles

The dimensions of the skull and lower jaw were analysed for 124 Pannonian root vole specimens.

Based on values from the coefficient of variability, the greatest variability was found in the adult population for the width of the neurocranium “LaN” and soft palate length “LBP”. The remaining measured dimensions all showed little variability. The tiniest variability was seen in soft palate width “PS” (Table 5).

The sub-adult population of the Pannonian root vole specimens had the greatest variability in the dimensions of the opening in the hard palate “FI”, the length of the upper teeth “LOSD”, and neurocranial width “LaN”. The smallest variability in cranial features was found in the length of protrusions in the neck (LM) and in the width of the soft palate “PS” (Table 6).

Among the juvenile Pannonian root vole population, the width of the neurocranium “LaN”, the length of the soft palate “LBP”, and the length of the facial part of the skull “LuV” showed the greatest variability in dimensions. The smallest cranial variability was seen in the width of the temporal bones “Io” and the length of the upper teeth “LOSD” (Table 6).

Lower teeth length “LOID” showed the greatest variability among the morphological lower jaw features in adult specimens (Table 7), while the smallest variability was in jaw length without the front teeth “ML”.

The greatest variability among sub-adult specimens was found in diastema length in the jaw “LMdD” while the smallest was in the length of the mandible “LMd”. In juvenile specimens, the greatest variability was documented in the length of the jaw without the front teeth “ML”, while the smallest variability was found in the length of the lower teeth “LOID” (Table 8).

**Figure 3:**
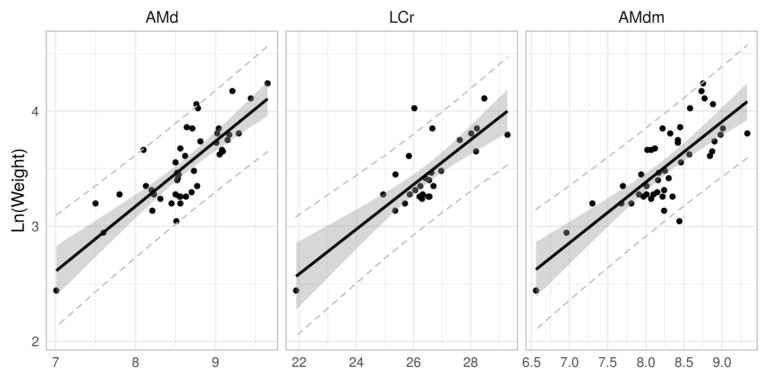
Relationship between weight and craniological data for Pannonian root vole with 95% confidence (grey area) and 95% prediction intervals (dashed lines).

We also analysed the relationship between weight and craniological features. Values derived from the length of craniological features were analysed from the ratio of explained variability to weight (Table 9). The coefficient of determination (R^2^) indicates the dependency to be relatively low. R^2^ values around and above 0.6 were only determined for mandible length “AMd”, maximum height of mandibula excluding coronoid process “AMdm” (*coronoid process*) and for the overall length of the skull “LCr” (distance between the *Akrokranion* and *Prosthion* points). These are shown in Figure 3.

## 4. Discussion

Our results point to changes in average body length of adults affected by the season in which they were captured and also the gender of the voles. The body length of specimens caught in autumn was demonstrably greater than those captured in spring. Kratochvil and Rosický [9] measured the body length of adult Pannonian root vole specimens (from the previous year’s litter) that had been caught in Slovakia during the summer and reported a range of 116 to 142 mm. Krištofík and Stollmann [14] likewise documented body lengths between 82 and 142 mm for specimens in Slovakia, but without classifying them by age. Bauer (1953) indicated an adult body length for the subspecies *mehelyi* in Austria falling between 112 and 138 mm (mean length of 121.1 mm). Éhik [12] likewise measured an adult body length for *mehelyi* in Hungary between 105 and 130 mm (mean length of 112.8 mm). Our results point to changes in average adult body length of adults affected by the season in which they were captured and also the gender of the voles. The body length of specimens caught in autumn was demonstrably greater than those captured in spring. Litters are born from early spring until autumn [6,9,31]. Body length increases from spring to autumn (or possibly into winter) and the specimens’ own growth naturally reflect it. Hulejová-Sládkovičová et al. [31] documented a subsequent slowdown in the growth of Pannonian root vole specimens from autumn into winter and conversely intensive growth in wintering specimens from winter to spring (females by 0.77 mm a week and males by 1.68 mm a week). In the next phase of the life cycle (from the following summer to autumn), some specimens exhibited a decline in body length, which the authors explained was by them ageing. Hulejová-Sládkovičová et al. [31] additionally wrote about Pannonian root voles not surviving two straight winters. The body length plays a critical role in them successfully overwintering, with a trade-off between the advantage of a large body for surviving cold winter conditions and the lower predation risk smaller animals enjoy along with them requiring shorter foraging times [32,33,34]. Based on our measurements and also data from Kratochvíl and Rosický [9], it appears that Slovakia’s Pannonian root vole population grows to a greater body length than populations in both Austria and Hungary.

Tail length of adults, sub-adults, and juveniles fall within the range 27 and 73.5 mm (which is consistent with the following two works), but the average tail length of adults is affected by both the season when they were captured and their gender. According to Kratochvil and Rosický [9], the Pannonian root vole is the subspecies with the longest tail. Bauer [11] also evaluates it as a long-tailed subspecies, while other authors have reported adult tail length to vary between 28 and 73 mm (for wintering voles, their tails range from 38 to 73 mm), with the tails of the largest specimens longer than half the body length [9]. Krištofík and Stollmann [14] also indicated tail lengths ranging from 28 to 72 mm, but without subdividing the specimens by age. Our results of adults, sub-adult and juveniles fall within the range both previous studies measures (in one case we found a 27 mm long tail in a young specimen), but the average tail length of adults is affected by both the season when they were captured and their gender. The effect of season and gender on sub-adult and juvenile specimens was not analysed due to a lack of data. Our results showed the ratio between mean tail length and mean body length not to have changed with age from the sub-adult to adult categories and for a stable ratio to have been maintained in terms of age and gender (i.e., the tail comprises of 45.12–46.82% of body length). This corresponds fully with the findings made by Kratochvil and Rosický [9] and highlights the taxonomical value of this feature.

Based on our results, the length of the hind foot ranges from 15.5 to 23 mm and 18.5–23 mm, respectively, although only for adult specimens. In the adult specimens, however, the average length of the hind foot was affected by gender. The authors noticed the length of the hind foot varying within an interval of 18.5 to 22.6 mm, with a range among the adult population between 19.5 and 22.4 mm [9]. Our results documented variability for all age categories within an interval of 15.5–23 mm and 18.5–23 mm, respectively, although only for adult specimens. In the adult specimens, however, the average length of the hind foot was affected by gender. On the other hand, stability in the length of the hind foot was shown across seasons (not affected by seasonality). This is in line with the assertion by Kratochvil and Rosický [9] that the length of the hind foot was a reliable determining feature.

Adult weights are from 20 to 69.5 g. Kratochvil and Rosický [9] reported body weight ranging from 23 to 61 g in specimens caught after they had overwintered. Our results showed average weight falling between summer and autumn. Both Krištofík and Stollmann [14] and Kratochvíl and Rosický [9] noticed a tendency for weight to decline from the growing season into autumn. Our results showed average weight falling between summer and autumn. This drop could be explained by specimens born later in the year accumulating energy in order to survive winter and then mating in the second year of their lives [35,36,37]. Specimens born later in the season grow slower and overwinter as sub-adults [35]. No change in average weight between seasons was documented for the adult voles in this investigation.

Because there are not enough references covering somatic traits of the subspecies *Alexandromys oeconomus mehelyi*, it was not possible to compare our findings with published data about Austrian and Hungarian populations.

Kratochvíl and Rosický [9] found the following cranial feature values for the *mehelyi* subspecies in Slovakia: LCB ranging from 26.3–30.5 mm, Io: 3.3–4 mm, LD: 8–10 mm, LOSD: 6.2–7 mm; Bauer [11] presented the following data in Austria: LCB ranging from 27.6–31.1 mm, Io: 3.7–4.1 mm, LD: 8.3–9.6 mm, LOSD: 6.9–7.3 mm; Éhik [12] presented the following data in Hungary: LCB ranging from 27–30 mm, Io: 3.5–4 mm, LD 7.9–9 mm, LOSD 6.4–7.1 mm. Based on the dimensions of cranial features, similar values can be said to have been reached among Austrian, Hungarian and Slovakian populations. Our craniometric results concur with the claim by Kratochvíl and Rosický [9], even though the Pannonian root vole is among the largest root vole sub-species.

Rácz et al. [13] morphologically analysed similarities in jaws and skulls in the historical relationships between Hungarian populations and found the root vole population in Hungary to be composed of four regional groupings. Two different northern groupings occupy the area around Szigetköz and Hanság. The third grouping comprised a population at Lake Balaton and the fourth highly divergent group was composed of specimens from the Kiskunság region of Hungary. The Hanság population shows the least morphological divergence compared to the other groupings, which indicates it either capable of supporting a greater root vole population or to be a dispersal centre for the colonisation of suitable habitats in Hungary.

Baláž and Fraňová [10] evaluated somatic and craniological biometrics among Pannonian root vole sub-species *Microtus oeconomus mehelyi* (Éhik, 1928) in Slovakia and *Microtus oeconomus stimmingi* (Nehring, 1899) from Poland. In all cases, the greatest variability was seen in body length and the least in the length of the hind feet. Higher mean body weight and length were displayed in Poland’s vole population but they also exhibited lower tail and hind foot lengths, which follows Bergmann’s and Allen’s rules. A positive correlation between weight and length was demonstrated in both sub–species. While a negative correlation was found between body and tail lengths in specimens measured in Poland, a positive correlation was noticed among specimens in Slovakia. Similar results were confirmed when the dependence between the length of the body and of the hind foot was tested. Nineteen craniological features were also tested. The *stimmingi* sub-species reached the highest average values in all but four of them (LFm, Ia, Io, LOSD). Of the six evaluated jaw features, higher mean values were found in the sub-species *mehelyi* for three of them, LMd, LMdD, AMd, and lower mean values for the remaining features. In the case of the *stimmingi* subspecies, the other observed features (Amdm, LOID, ML, USA) had higher mean values.

The relationship between weight and craniological data was analysed by us. Based on the coefficient of determination (R^2^), a relatively low dependency was found. This may be due to the assumption of continuous growth in body mass and bone inherent in the linear regression. In the case of mammals, there may be a disproportion between the rate of cranial bone growth and either the specimen’s weight or overall size. The craniological features considered by us to be the most appropriate for estimating weight from skeletal remains are mandible length “AMd” maximum height of mandibula excluding coronoid process “AMdm” (*coronoid process*), and for the overall length of the skull “LCr” (distance between the Akrokranion and Prosthion points). Calculating the weight from cranial remains can have practical applications, such as to estimate prey size and determine the biomass of small mammals that have been consumed by raptors, owls, or other predators.

The relationship between rodent body weight and age, and also cranial and body measurements, was analysed several decades ago [15,16], where they discovered significant correlation between different craniometric measurements and the body weight of small mammals [17,18,19]. Regression equations have been suggested as instruments to measure the body weight of small mammals consumed by various predators [22,23,24,25,26]. Pagels and Blem [16] estimated small mammal body weight from the dimensions of their skulls. They found the best predictive equations obtained from cranial measurements usually contained three independent variables that varied between species. Equations involving measurement of just mandibular length were less accurate than those based on cranial measurements. To obtain maximum accuracy from predictive equations, the sample size should be at least 40 specimens. Blem et al. [23] calculated the body weight of voles (*Microtus pennsylvanicus*, *M. montanus*) that had been discarded by short-eared owls (*Asio flammeus*). They found regression to be routinely better for estimating body weight from cranial dimensions, provided all prey specimens are adults, and when calculating weight from individual skeletal measurements. Balčiauskienė and Balčiauskas [26] wrote that regression equations derived from cranial measurements can predict the body weight of bank voles, which explained 38–58% of body weight variability. They found three mandibular features—LMd, ML, and AMdm—and three maxillary features—LaZ, LD, and FI—to correlate better to bank vole body weight. The accuracy of the predictions derived from the regressions was very high and the error in predicting body weight ranged between 1.2% and 4.4%.

Balčiauskas and Balčiauskiené [27] opted to estimate the body weight of *Microtus oeconomus stimmingi* from 26 cranial and pelvic features. The correlation between 20 of the measured features and body weight was strong (r ≥ 0.6, *p* < 0.0001). Seven linear and multiple regressions estimated body weight with an error in the range of 5.5–15.0% from actual, measured body weight. Such a difference was not statistically significant in the study of Balčiauskas and Balčiauskiené.

To conclude, somatic features indicate that specimens caught in Slovakia, specifically in the northern Pannonian Plain, belong to the largest sub-species *oeconomus*. Overall, our results showed gender to have an impact on the mean values of all the somatic features observed in adult specimens, while the effect of seasonality was only seen in the average length of the body and tail. Thus, mean body and foot length appear stable in determining features despite the seasons. Our study also evaluated 25 skull and lower jaw dimensions from the largest material that has been collected to date, representing the largest number of evaluated characteristics among Slovakia’s Pannonian root vole population. Linear regression of the weight and any of the three craniological features AMd, AMdm, and LCr are recommended in order to predict weight of voles directly from them. This analysis is useful as a non-invasive method for analysing skeletal food remains that have been found in raptors and owls. Subsequently using the correlation between weight and body length makes it possible to analyse the Pannonian root vole population structure in greater detail, such as to classify specimens into age cohorts [31]. Because the species has become rare and is now highly endangered, together with the ongoing lack of scientific knowledge, any information about the biology and ecology of the Pannonian root vole contributes toward its protection and survival in the future.

## Figures and Tables

**Table 1:** Overview of Pannonian root vole specimens morphometrically analysed.

Sex	Age Group				Total
	Adults	Sub-Adults	Juveniles	Unspecified	
**Somatometry**					
**Males**	121	29	8	-	158
**Females**	117	65	15	-	197
**Total**	238	94	23	-	355
**Craniometry**					
**Males**	36	11	3	-	50
**Females**	35	16	7	-	58
**Unspecified**	-	-	-	16	16
**Total**	71	27	10	16	124

**Table 2:** Somatic characteristics of Pannonian root vole.

Group	Somatic Characteristics	*n*	Average ± SD	CV (%)	Range
**Adults**	body weight (g)	231	41.56 ± 10.15	24.4	20–69.5
	body length (mm)	107	119.26 ± 10.23	8.58	97–154
	tail length (mm)	105	54.43 ± 7.33	13.47	38–73.5
	length hind foot (mm)	112	20.25 ± 0.96	4.73	18.5–23
**Adult males**	body weight (g)	119	43.1 ± 9.94	23.06	23–68
	body length (mm)	52	121.36 ± 10.31	8.49	52–104
	tail length (mm)	50	56.09 ± 7.65	13.63	43–73.5
	length hind foot (mm)	54	20.48 ± 0.99	4.84	19–23
**Adult females**	body weight (g)	112	39.74 ± 10.6	26.67	25–69.5
	body length (mm)	55	117.28 ± 9.85	8.39	87–123
	tail length (mm)	55	52.92 ± 6.75	12.76	38–64.5
	length hind foot (mm)	58	20.03 ± 0.88	4.39	18.5–22
**Sub-adults**	body weight (g)	90	25.7 ± 4.36	17.49	15–35
	body length (mm)	37	99.84 ± 6.49	6.49	85.5–117
	tail length (mm)	37	45.85 ± 5.95	12.97	34–70
	length hind foot (mm)	38	20.03 ± 0.77	3.85	18–21
**Sub-adult males**	body weight (g)	27	25.12 ± 4.5	17.94	16–35
	body length (mm)	15	101.67 ± 6.14	6.05	90–117
	tail length (mm)	15	47.6 ± 3.38	7,09	42–55
	length hind foot (mm)	15	20.1 ± 0.71	3.54	19–21
**Sub-adult females**	body weight (g)	63	26.04 ± 4.51	17.33	15–35
	body length (mm)	22	98.59 ± 6.55	6.64	85.5–106
	tail length (mm)	22	44.66 ± 7.03	15.73	34–70
	length hind foot (mm)	23	19.98 ± 0.82	4.09	18–21
**Juvenile males**	body weight (g)	8		14.63	10–20
	body length (mm)	5		79.2	60–95
	tail length (mm)	6		35	27–42
	length hind foot (mm)	6		17.75	15.5–19.5
**Juvenile females**	body weight (g)	15		15.63	7–20
	body length (mm)	4		90.88	90–92.5
	tail length (mm)	5		37.9	34–40.5
	length hind foot (mm)	7		19.14	16.5–20

Explanations: *n*—Number of individuals, SD—standard deviation, CV—coefficient of variation.

**Table 3:** Two-way ANOVA analysis of the impact on four somatic characteristics of adult Pannonian root voles of season, sex, and the interaction between them.

**(Log) Body Weight**		**df _1, 212_**	***F-* Value**	***p***
	Season	2	0.84	0.433
	Sex	1	7.76	0.005 **
	Season * sex	2	2.69	0.07
**body length**		**df _1, 81_**	***F*-Value**	***p***
	season	2	5.32	0.007 **
	Sex	1	7.38	0.008 **
	Season * sex	2	0.40	0.669
**(log) tail length**		**df _1, 82_**	***F*-Value**	***p***
	season	2	11.00	<0.001 ***
	Sex	1	6.64	0.012 **
	Season * sex	2	0.06	0.943
**(log) length hind foot**		**df _1, 80_**	***F*-Value**	***p***
	season	2	0.64	0.530
	Sex	1	7.16	0.009 **
	Season * sex	2	0.73	0.483

Statistically significant differences at * *p* < 0.05, ** *p* < 0.01 and *** *p* < 0.0001.

**Table 4:** Pearson correlation table between four somatic features.

	Body Weight	Body Length	Tail Length	Length Hind Foot
**Body weight**	-	***	***	***
**Body length**	0.871	-	***	**
**Tail length**	0.717	0.771	-	**
**Length hind foot**	0.514	0.382	0.446	-

Statistically significant differences at ** *p* < 0.01, *** *p* < 0.0001.

**Table 5:** Cranial signs in adult Pannonian root vole.

Skull Features	Total Adults			Males		Females	
	*n*	Average ± SD (CV)	Range	*n*	Average ± SD	*n*	Average ± SD
**LCr**	51	26.97 ± 1.4 (5.18%)	23.35–29.98	29	27.09 ± 1.51	22	26.81 ± 1.25
**LCB**	49	26.36 ± 1.4 (5.31%)	22.56–29.14	28	26.47 ± 1.49	21	26.22 ± 1.28
**LB**	48	25.56 ± 1.44 (5.63%)	22.0–28.35	27	25.75 ± 1.56	21	25.31 ± 1.26
**LBP**	52	10.13 ± 0.98 (9.72%)	8.2–11.89	28	10.41 ± 0.89	24	9.81 ± 1.00
**LPm**	57	15.29 ± 0.79 (5.18%)	13.48–17.19	31	15.28 ± 0.81	26	15.29 ± 0.78
**LFm**	49	19.59 ± 1.27 (6.48%)	15.73–22.18	28	19.49 ± 1.32	21	19.71 ± 1.23
**LuV**	71	8.84 ± 0.59 (6.76%)	7.33–10.3	36	8.91 ± 0.68	35	8.76 ± 0.49
**LN**	71	7.75 ± 0.64 (8.21%)	6.45–9.21	36	7.87 ± 0.71	35	7.62 ± 0.54
**LOSD**	71	6.95 ± 0.4 (5.79%)	5.8–7.7	36	6.94 ± 0.44	35	6.97 ± 0.36
**LD**	71	8.26 ± 0.56 (6.77%)	6.29–9.45	36	8.27 ± 0.54	35	8.26 ± 0.59
**LM**	51	7.7 ± 0.45 (5.88%)	6.85–8.87	28	7.63 ± 0.46	23	7.79 ± 0.42
**FI**	71	4.73 ± 0.37 (7.9%)	3.79–5.66	36	4.69 ± 0.39	35	4.75 ± 0.35
**LaN**	51	10.55 ± 1.45 (13.79%)	8.42–13.13	27	10.01 ± 1.29	24	11.15 ± 1.41
**LOC**	52	8.53 ± 0.41 (4.79%)	7.6–9.78	29	8.57 ± 0.46	23	8.48 ± 0.34
**lS**	71	3.26 ± 0.27 (8.36%)	2.68–3.87	36	3.26 ± 0.30	35	3.26 ± 0.24
**PS**	71	5.1 ± 0.19 (3.75%)	4.55–5.6	36	5.06 ± 0.19	35	5.15 ± 0.19
**LaZ**	67	14.61 ± 0.78 (5.34%)	13.46–16.65	34	14.65 ± 0.83	33	14.57 ± 0.73
**Ia**	71	6.41 ± 0.55 (8.62%)	5.5–7.86	36	6.51 ± 0.53	35	6.31 ± 0.57
**Io**	70	3.71 ± 0.19 (5.29%)	3.13–4.01	36	3.72 ± 0.18	34	3.71 ± 0.21

*n*—Number of individuals, SD—standard deviation, CV—coefficient of variation (%).

**Table 6:** Cranial signs in sub-adult and juvenile Pannonian root vole.

Skull Features	Sub-Adults			Juveniles		
	*n*	Average ± SD (CV)	Range	*n*	Average ± SD (CV)	Range
**LCr**	24	25.23 ± 1.39 (5.54%)	22.6–28.29	7	23.16 ± 1.85 (8.0%)	20.8–25.38
**LCB**	24	24.54 ± 1.39 (5.67%)	22.12–27.79	6	22.32 ± 2.16 (9.69%)	19.33–24.39
**LB**	24	23.75 ± 1.36 (5.72%)	21.31–27.36	6	21.56 ± 2.16 (10.0%)	18.88–23.86
**LBP**	24	9.45 ± 0.71 (7.55%)	8.24–11.12	6	8.48 ± 0.96 (11.32%)	7.0–9.45
**LPm**	26	14.30 ± 0.84 (5.89%)	12.97–16.5	9	12.87 ± 1.33 (10.3%)	10.58–14.4
**LFm**	24	18.54 ± 0.95 (5.12%)	16.82–20.39	7	17.43 ± 1.48 (8.49%)	15.03–19.14
**LuV**	26	8.48 ± 0.69 (8.1%)	6.66–10.1	9	7.27 ± 0.81 (11.07%)	5.83–8.34
**LN**	26	7.55 ± 0.66 (8.73%)	5.92–8.95	9	6.33 ± 0.60 (9.5%)	5.4–7.32
**LOSD**	26	6.89 ± 0.73 (10.56%)	5.9–9.98	10	6.39 ± 0.24 (3.7%)	6.0–6.8
**LD**	26	7.65 ± 0.57 (7.56%)	6.36–9.00	10	6.99 ± 0.76 (10.82%)	5.85–7.96
**LM**	24	7.07 ± 0.29 (4.1%)	6.32–7.69	7	6.62 ± 0.52 (7.89%)	5.9–7.33
**FI**	26	4.53 ± 0.60 (13.29%)	3.76–6.95	10	3.85 ± 0.39 (10.38%)	3.19–4.56
**LaN**	24	9.59 ± 0.94 (9.74%)	8.58–13.08	7	9.2 ± 1.35 (14.68%)	7.14–10.81
**LOC**	24	8.39 ± 0.39 (4.65%)	7.69–9.25	6	7.62 ± 0.74 (9.78%)	6.65–8.46
**lS**	26	3.11 ± 0.24 (7.67%)	2.67–3.75	10	2.89 ± 0.31 (10.73%)	2.44–3.3
**PS**	26	4.95 ± 0.20 (4.14%)	4.48–5.44	10	4.89 ± 0.28 (5.8%)	4.35–5.14
**LaZ**	25	13.29 ± 0.91 (6.87%)	10.08–14.67	9	12.17 ± 0.77 (6.31%)	11.18–13.06
**Ia**	26	6.18 ± 0.61 (9.88%)	5.02–7.37	9	5.61 ± 0.44 (7.77%)	5.02–6.62
**Io**	25	3.64 ± 0.17 (4.69%)	3.37–4.05	9	3.57 ± 0.08 (2.17%)	3.45–3.69

*n*—Number of individuals, SD—standard deviation, CV—coefficient of variation (%).

**Table 7:** Adult jaw-bone characteristics of Pannonian root vole.

Jawbone Features	Adults			Males		Females	
	*n*	Average ± SD (CV)	Range	*n*	Average ± SD	*n*	Average ± SD
**LMd**	70	15.31 ± 0.87 (5.7%)	11.96–17.48	35	15.3 ± 1.00	35	15.31 ± 0.73
**LMdD**	70	4.43 ± 0.29 (6.69%)	3.42–5.15	35	4.44 ± 0.32	35	4.42 ± 0.27
**LOID**	70	6.84 ± 0.54 (7.87%)	5.19–8.07	35	6.91 ± 0.62	35	6.77 ± 0.44
**ML**	68	14.98 ± 0.81 (5.43%)	12.1–17.16	34	14.92 ± 0.96	34	15.04 ± 0.64
**AMdm**	70	8.43 ± 0.50 (5.95%)	6.64–9.84	35	8.42 ± 0.58	35	8.45 ± 0.42
**AMd**	69	8.72 ± 0.51 (5.86%)	7.09–9.65	34	8.68 ± 0.58	35	8.75 ± 0.44

*n*—Number of individuals, SD—standard deviation, CV—coefficient of variation (%).

**Table 8:** Morphometric data for jaw-bone dimensions of sub-adult and juvenile Pannonian root vole.

Jawbone Features	Sub-Adults			Juveniles		
	*n*	Average ± SD (CV)	Range	*n*	Average ± SD (CV)	Range
**LMd**	26	14.61 ± 0.83 (5.68%)	12.8–16.59	10	13.31 ± 1.09 (8.26%)	11.63–14.35
**LMdD**	26	4.20 ± 0.35 (8.32%)	3.57–4.92	10	4.05 ± 0.37 (9.23%)	3.44–4.77
**LOID**	26	6.70 ± 0.46 (6.79%)	5.7–7.8	10	6.27 ± 0.38 (6.0%)	5.75–6.95
**ML**	26	14.22 ± 0.84 (5.88%)	12.44–16.7	10	12.99 ± 1.24 (9.56%)	11.2–14.25
**AMdm**	26	7.77 ± 0.55 (7.14%)	6.6–9.21	10	6.79 ± 0.59 (8.83%)	5.81–7.63
**AMd**	25	7.99 ± 0.53 (6.62%)	6.94–8.94	10	7.20 ± 0.58 (8.08%)	6.17–7.99

*n*—Number of individuals, SD—standard deviation, CV—coefficient of variation (%).

**Table 9:** Craniological features ordered by ratio of explained variability to the log-transformed weight of Pannonian root vole. To predict weight, use: e^α + β*x^ where *x* is measured variable value.

Variable	Coefficient of Determination (R2)	*p*-Value	Intercept (α)	Slope (β)	% Increase of Weight per 1 mm of Variable
**AMd**	0.64826241	0.0000000	−1.349462	0.565424955	76.02
**LCr**	0.632964852	0.0000007	−1.6781435	0.193871582	21.39
**AMdm**	0.597003542	0.0000000	−0.8290947	0.526504854	69.30
**LFm**	0.57965912	0.0000099	−1.4066505	0.250263025	28.44
**LaZ**	0.578798151	0.0000000	0.0433793	0.244496822	27.70
**LMd**	0.539016837	0.0000000	−1.5542477	0.337277443	40.11
**ML**	0.506645887	0.0000001	−3.3236193	0.46186331	58.70
**LCB**	0.432548489	0.0004783	−0.8646062	0.167782622	18.27
**LPm**	0.422301147	0.0000761	−1.2187047	0.31547443	37.09
**LD**	0.421328611	0.0000019	−0.1286731	0.450949592	56.98
**LB**	0.396151461	0.0012916	−0.4180592	0.155491846	16.82
**LBP**	0.3407932	0.0013901	1.5655657	0.198865391	22.00
**FI**	0.320092342	0.0000627	0.7089643	0.618912659	85.69
**LMdD**	0.239028045	0.0007585	0.6883105	0.646249598	90.84
**LOSD**	0.236503192	0.0008169	−0.4695393	0.578412291	78.32
**Ia**	0.234984159	0.0008541	1.4858656	0.319414852	37.63
**LuV**	0.216666842	0.0014529	1.1132867	0.277396959	31.97
**PS**	0.185287622	0.0035387	−1.5812038	0.999713419	171.75
**LN**	0.176366142	0.0045394	1.6440319	0.247687397	28.11
**LOID**	0.155197508	0.0081475	1.2543411	0.335258091	39.83
**IS**	0.123816592	0.0191629	2.0756368	0.44052701	55.35
**LM**	0.100883386	0.0931516	2.1218833	0.181239785	19.87
**Io**	0.084364277	0.0588277	1.5444953	0.527866793	69.53
**LOC**	0.072926639	0.1565495	1.50637	0.241622055	27.33
**LaN**	0.001120023	0.8683806	3.4355023	0.007856462	0.79

## Data Availability

The study did not report any data.

## Supplementary Materials

The following are available online at https://www.mdpi.com/2076-2615/11/2/576/s1, Table S1: Biometrically analysed specimens of Pannonian root vole.
